# Supplementary material for: Identification of the needs of children with neurodisability and their families at different stages of development: A qualitative study protocol
Source: PLoS One. 2023 Sep 8;18(9):e0291148. doi: 10.1371/journal.pone.0291148 (PMC10490905; doi:10.1371/journal.pone.0291148)
Supplement: S1 Appendix — (PDF) [file pone.0291148.s001.pdf]

S1 Appendix. Satisfaction Survey.

1. Rate your overall experience after participating in the focus groups, assigning a score from 0 (very dissatisfied) to 5 (very satisfied).

0	1	2	3	4	5
○	○	○	○	○	○

2. Rate specifically your experience in the different topics discussed in the focus group, assigning a score from 0 (very dissatisfied) to 5 (very satisfied).

	0	1	2	3	4	5
Function	○	○	○	○	○	○
Fitness	○	○	○	○	○	○
Family	○	○	○	○	○	○
Friends	○	○	○	○	○	○
Fun	○	○	○	○	○	○
Future	○	○	○	○	○	○

3. Do you feel that the focus group has addressed the different stages of your child's development (early childhood, infancy, pre-adolescence, adolescence) beyond motor or verbal skills according to his/her age?

☐ Yes, I consider that my child's stage has been fundamentally addressed.

☐ Both aspects (developmental stage and motor/verbal ability) have been addressed.

☐ I only consider that motor and/or verbal ability has been discussed.

☐ I do not agree with any of the above answers.

4. In case you indicated "I do not agree with any of the above answers" in the previous question, we invite you to write your opinion below.

Please,write your answer

5. Do you have any other comments you would like to share?

Please,write your answer
