# Supplementary material for: Identification of the needs of children with neurodisability and their families at different stages of development: A qualitative study protocol
Source: PLoS One. 2023 Sep 8;18(9):e0291148. doi: 10.1371/journal.pone.0291148 (PMC10490905; doi:10.1371/journal.pone.0291148)
Supplement: S2 Appendix — (PDF) [file pone.0291148.s002.pdf]

S2 Appendix. Sociodemographic and Clinical Data Survey.

1. E-mail address of parent/guardian.

Please, enter your response

2. Which member/s of the family will participate in the focus group?

☐ Father

☐ Mother

☐ Legal guardian

☐ Child (if age is between 12 and 18 years old)

3. Name and surname of Parent/Guardian 1.

Please, enter your response

4. Age of Parent/Guardian 1.

Specify date (dd/MM/yyyy)

5. Occupation of Parent/Guardian 1.

Please, enter your response

6. Name and surname of Parent/Guardian 2.

Please, enter your response

7. Age of Parent/Guardian 2.

Specify date (dd/MM/yyyy)

8. Occupation of Parent/Guardian 2.

Please, enter your response

9. Name of your child.

Please, enter your response

10. Your child's date of birth

Specify date (dd/MM/yyyy)

11. Can you tell us what diagnosis your child has?

Please, enter your response

12. Type of schooling:

☐ Ordinary school.

☐ Ordinary school with preferential attention to motor skills.

☐ Special education school.

☐ Other.

13. If you indicated "other" in the previous question, please specify below the type of schooling.

Please, enter your response

14. In case your child is taking any medication, can you please indicate the name of the medication and the daily dosage?

Please, enter your response

15. What kind of intervention/s does your child receive either inside or outside the school environment?

☐ Physiotherapy

☐ Occupational therapy

☐ Speech therapy

☐ Neuropsychology

☐ Other

16. If you have indicated "other" in the previous question, please specify below the type of interventions your child receives.

Please, enter your response

17. Can you tell us the weekly frequency of the interventions?

Please, enter your response

18. Which positioning systems does your child use? (Single option).

☐ Standing frame.

☐ Pelvic moulded seat.

☐ None.

☐ Other.

19. In case you indicated "other" in the previous question, please specify below the positioning system your child uses.

Please, enter your response

20. Which mobility aids does your child use?

☐ Crutches or cane.

☐ Rear walker.

☐ Front walker.

☐ Manual wheelchair.

☐ Electric wheelchair.

☐ None.

☐ Other.

21. If you have indicated "other" in the previous question, please specify below which mobility aid your child uses.

Please, enter your response

22. Is your child a user of ankle-foot orthoses?

☐ YES

☐ NO

☐ My child was in the past but no longer uses it.

23. Describe what the ankle-foot orthosis is/was like (high, low, if it has a tibial strap, if it is hinged...).

Please, enter your response

24. Have you received prior advice on how to implement positioning systems, mobility aids or foot-ankle orthoses into routines?

Please, enter your response

25. We would like to know what extracurricular activities your child does. Can you tell us what activities your child does outside school and how often per week?

Please, enter your response

26. If there are siblings in the family unit, can you tell us the age of each of the siblings?

Please, enter your response

27. In your daily life, what resources do you use to obtain information about the pathology you live with?

☐ Internet (Internet search engines such as Google).

☐ Associations of families or patients with the same pathology.

☐ Scientific journals/scientific papers.

☐ Professionals around you.

☐ Others.

28. If in the previous question you indicated "other", please specify below the resources you use to obtain information about the pathology you are living with.

Please, enter your response
