# Supplementary material for: Identification of the needs of children with neurodisability and their families at different stages of development: A qualitative study protocol
Source: PLoS One. 2023 Sep 8;18(9):e0291148. doi: 10.1371/journal.pone.0291148 (PMC10490905; doi:10.1371/journal.pone.0291148)
Supplement: Attachment — Submitted filename: Response to Reviewers_R1.docx [file pone.0291148.s003.docx]

**Prof. Renato S. Melo**

**Academic Editor of PLOS ONE**

July 14^th^, 2023

Dear Prof. Renato S. Melo,

Thank you for the opportunity to revise our manuscript, “*Identification of the needs of children with neuromotor pathology and their families at different stages of development: a qualitative study protocol*” (PONE-D-23-10164). We are grateful for the time and effort that the editorial team and the reviewers have put into the response, which we feel has helped us improve the manuscript. Our point-by-point response has addressed all comments and suggestions, and we have changed the manuscripts accordingly. All responses and changes have been highlighted using a **blue font** for easier tracking in the tracked manuscript version. Furthermore, we attach a clean version of the revised manuscript.

We hope you and the reviewers find our responses to your satisfaction, and we look forward to hearing from you.

Yours sincerely,

Pablo Bellosta-López, PhD

Universidad San Jorge

Campus Universitario, Autov. A23, Km 299, 50830 Villanueva de Gállego, Zaragoza, Spain

Tlf. (+34) 976 060 100

Fax (+34) 976 077 581

[pbellosta@usj.es](mailto:pbellosta@usj.es)

Reviewer(s)' Comments to Author:

**Reviewer: 1**

**Review Comments to the Authors**

Thank you for the opportunity to review the manuscript. Indeed, the topic is relevant. However, I just have one question. In the inclusion criteria. Neuromotor pathology at pediatric age... what are they in fact?

Authors: Thank you very much for your contribution. We also find your comment very useful.

The population we are addressing is under the scope of the term neurodisability. This is considered an umbrella term that includes pathologies related to intellectual disability, Down syndrome, cerebral palsy, spina bifida, and autism. Since it is a very broad term, we tried to simplify it with the term neuromotor pathology. However, after your comment, we have changed this term to neurodisability, as indicated in the literature.

From: “neuromotor pathology” throughout the entire manuscript

To: “neurodisability”

We also include the definition of the term and adapt the initial paragraphs of the introduction to clarify to which population it refers. A reference to this is added. (page 3, line 47- 57).

From: “Neuromotor pathology in childhood causes disability either during a particular stage of development or permanently [1]. Traditionally, medical efforts have focused on diagnosing and treating diseases and chronic developmental disorders, with less attention paid to the functional capacities of individuals and their contextual determinants [2]. A key social determinant is family, as parents are the main support providers for children with neurodisabilities at all stages of development [3]. Each of these stages shows different circumstances for each family unit, which are unique and different in functioning, behavior, and context [4].”

To: “Neurodisability is an umbrella term attributed to brain or neuromuscular system impairments characterized by functional limitations and difficulties with movement, cognition, hearing and vision, communication, emotion, and behavior [1]. Traditionally, medical efforts have focused on diagnosing and treating chronic developmental disorders that cause neurodisability (e.g., intellectual disability, Down syndrome, cerebral palsy, spina bifida, and autism spectrum disorder [1]) from a biomedical perspective, with less attention paid to the functional capacities of individuals and their contextual determinants [2]. However, these aspects are important, especially in the management of childhood neurodisability [3], as parents are the main support providers for children with neurodisabilities at all stages of development [4]. Furthermore, each of these stages is unique and different in functioning, behavior, and context for each family unit [5].”

1.	Meehan E, Gallagher AL, Ryan J, Kerr C, R OS, Galvin R, et al. Unmet healthcare needs in adults with childhood-onset neurodisabilities: a protocol for a systematic review. HRB Open Res. 2021;4:107. Epub 2022/09/09. doi: 10.12688/hrbopenres.13309.1. PubMed PMID: 36071876; PubMed Central PMCID: PMCPMC9399657.

**Reviewer: 2**

**Comments and Suggestions for Authors**

I congratulate the authors for the effort in promoting current recommendations for research with children that have disabilities and their families. However, I believe the manuscript quality should be improved. I provide some suggestions next.

Overall: I feel that the authors could improve the study rationale by justifying why considering the classification levels is not sufficient (Perhaps at younger ages a prognosis is not clear? ) and why differences are expected across ages.

Also, perhaps not many studies have considered different developmental stages at once, but many studies have looked into specific age-ranges. A comprehensive literature overview is lacking, as well as a clear gap to be addressed.

Examples:

- Byrne, R., Duncan, A., Pickar, T., Burkhardt, S., Boyd, R. N., Neel, M. L., & Maitre, N. L. (2019). Comparing parent and provider priorities in discussions of early detection and intervention for infants with and at risk of cerebral palsy. Child: Care, Health and Development, 45(6), 799-807.
- Graungaard, A. H., & Skov, L. (2007). Why do we need a diagnosis? A qualitative study of parents’ experiences, coping and needs, when the newborn child is severely disabled. Child: care, health and development, 33(3), 296-307.
- Resch, J. A., Mireles, G., Benz, M. R., Grenwelge, C., Peterson, R., & Zhang, D. (2010). Giving parents a voice: A qualitative study of the challenges experienced by parents of children with disabilities. Rehabilitation psychology, 55(2), 139.

Authors: We really appreciate this comment. We totally agree that there was a lack of explanation regarding the rationale and justification of investigating families´ needs through ICF and F-words at each stage of the development. We have made changes throughout the Introduction to better clarify this aspect.

“Family-centered care is considered the best practice when providing services to children with neurodisability [6] and is associated with better outcomes based on meaningful goals set by children with neurodisabilities and their families. An essential aspect of family-centered care is the provision of resources, support, and services in response to family-identified needs and priorities [7]. However, families face significant challenges in identifying their personal, social, service-related, disability-related, and financial needs. In turn, unclear families' needs make children care even more challenging, as it might worsen pre-existing barriers such as the lack of proper access to information and environmental support [8].

…

For the design of actions that promote such empowerment, different studies have investigated the needs of families in different contexts, whether to understand the profile of families, the provision of necessary services, or financial and community support [16-19]. These studies have considered the level of gross motor function or the level of communication to categorize the needs. However, none of these studies has delved into the needs of children with neurodisability and their families within the framework of the F-words in the different stages of development, not only establishing the categories according to motor and/or verbal ability but according to the vital stage of development of each child. Investigating families´ needs through the F-words at each stage of the children development at once is an innovative approach that can help (i) providing families with more accurate and tailored information regarding e.g., prognosis or severity, during early diagnostic process [20], which in turn could make families to cooperate more actively with the situation [21], (ii) observing potential differences across development stages and (iii) reducing the mismatch between families´ needs, healthcare services and policy-making [8].

It is therefore necessary to give a voice to children with neurodisability and their families through the lens of ICF/F-words at different stages of development. Thus, they can identify and express their experience regarding vital aspects of their daily lives that interfere with the holistic development of children in their different stages.”

8.	Resch JA, Mireles G, Benz MR, Grenwelge C, Peterson R, Zhang D. Giving parents a voice: A qualitative study of the challenges experienced by parents of children with disabilities. Rehabil Psychol. 2010;55(2):139-50. Epub 2010/05/26. doi: 10.1037/a0019473. PubMed PMID: 20496968.

20.	Byrne R, Duncan A, Pickar T, Burkhardt S, Boyd RN, Neel ML, et al. Comparing parent and provider priorities in discussions of early detection and intervention for infants with and at risk of cerebral palsy. Child Care Health Dev. 2019;45(6):799-807. Epub 2019/07/20. doi: 10.1111/cch.12707. PubMed PMID: 31323144.

21.	Graungaard AH, Skov L. Why do we need a diagnosis? A qualitative study of parents' experiences, coping and needs, when the newborn child is severely disabled. Child Care Health Dev. 2007;33(3):296-307. Epub 2007/04/19. doi: 10.1111/j.1365-2214.2006.00666.x. PubMed PMID: 17439444.

Authors: Furthermore, we have expanded the Discussion section with updated references (page 16, line 360-363).

“… Likewise, there are innovative studies that propose training programs for parents of children with neurodisabilities (0-6 years of age) that aim to provide information, support and preparation tools for the future and to get their opinion on the matter [56]; however, have not yet been applied to other developmental stages.”

56.	Miller L, Imms C, Cross A, Pozniak K, O'Connor B, Martens R, et al. Impact of "early intervention" parent workshops on outcomes for caregivers of children with neurodisabilities: a mixed-methods study. Disability and rehabilitation. 2022:1-12. Epub 2022/11/22. doi: 10.1080/09638288.2022.2143579. PubMed PMID: 36404703.

page 4

When listing the F-words and respective ICF components, the authors missed the F-word Function.

Authors: Thank you for your input. We have clarified the information. (page 4, line 76).

From: “Therefore, the F-words function relates to the activity…”

To: “Therefore, the F-word “Function” relates to the activity…”

Objectives:

2- Even though they are related, ICF components and F-words are not synonyms. Please adjust accordingly.

Authors: We agree with you and appreciate the suggestion. We clarify objective 2 following your recommendation. (page 5, line 117).

From: “To assess whether the needs detected are susceptible to being categorized within the conceptual framework of the ICF (i.e., F-words).”

To: “To assess whether the needs detected are susceptible to being categorized within the conceptual framework of the ICF through the resource provided by the F-words”

2 and 3- I do not see how these objectives fit the study rationale. Please clarify.

Authors: The rationale provides three pillars that are connected when talking about neurodisability. One of the pillars is family-centered practices; the second is ICF through the F-words resource, and the third is family empowerment.

Objective 2 aims to categorize the needs through the F-words framework in order to make them visible to society. On the other hand, objective 3 establishes the need to define the family context through socio-demographic data, essential information when talking about family-centered practices, and family empowerment.

It is considered essential to address all the pillars, knowing the needs as well as the family context in which they occur.

We hope to have clarified your comment. Thank you.

Methods:

Please add information about study registration if applicable.

Authors: In this case, the study does not require registration. Thank you for your contribution.

Procedures: Will participants receive any previous instructions on the f-words framework? If so, how they will be provided?

Authors: Thank you for your contribution. This information will be included in the participant information sheet. It will be also explained by the principal investigator when contacting the potential participants.

Therefore, the following information is added. (page 7, line 169).

From: “After contacting the principal investigator, reading the information, and asking any questions,…”

To: “After contacting the principal investigator, reading the information (which includes instructions on the F-words framework), and asking any questions,…”

Data analyses: It is not clear if the authors will follow methods as recommended by the WHO for linking concepts to ICF (e.g., linking rules).

Authors: We would like to thank the reviewer for this comment, as the use of the ICF linkage is our proposed question guide, but not our model of analysis. We like the idea of making this linkage in the future (perhaps in the discussion).

That is, unlike other authors who look for the ICF linkage in the content analysis (such as A.C. MacDermid in his 2021 article), we want the themes that emerge after the thematic analysis to do so in a completely inductive way, far from the questions of the semi-structured guide itself.

So, even if we ask about the F-words, the themes may be about emotional management by parents, resourcing by professionals, or lack of resourcing by institutions. We agree that they may be similar to the contextual factors of the ICF but reported from a more inductive point of view. (page 11 line 255- 261)

From: “In the initial stages of coding and analysis, the level of abstraction of the data will be more descriptive. Progressively, the complexity and level of abstraction will increase to identify the essence. Thus, the analysis in the last phases will be more interpretative, assessing whether the themes and sub-themes obtained are susceptible to being categorized within the conceptual framework of the ICF (F-words).”

To: “In the initial stages of coding and analysis, the level of abstraction of the data will be more descriptive. Progressively, the complexity and level of abstraction will increase to identify the essence. Thus, the analysis in the last phases will be more interpretative. In this thematic analysis proposal, a fully inductive analysis will be carried out, in which themes will emerge after the groupings of Units of meaning and common Groups of Meaning. Measure to ICF linkage, Measure to (Brief or Comprehensive) Core Set Absolute Linkage, Measure to (Brief or Comprehensive) Core Set Unique Linkage, Core Set Representation, and Core Set Unique Disability Representation indicators will not be used during the analysis but will be taken into account to relate concepts in the discussion after analysis of the results.”
